# Ethical considerations during Mpox Outbreak: a scoping review

**DOI:** 10.1186/s12910-024-01078-0

**Published:** 2024-07-22

**Authors:** Fatma Badr El Dine, Assem Gebreal, Dalia Samhouri, Haimanot Estifanos, Islam Kourampi, Hasnaa Abdelrhem, Hamed Abdelma’aboud Mostafa, Ahmed Gamal Elshaar, Tarun Kumar Suvvari, Ramy Mohamed Ghazy

**Affiliations:** 1 Forensic Medicine and Clinical Toxicology, Faculty of Medicine, Alexandria University, Alexandria, Egypt; 2 Faculty of Medicine, Alexandria University, Alexandria, Egypt; 3 Regional Manager, Emergency Preparedness & International Health Regulations WHO Regional Office for the Eastern Mediterranean, Cairo, Egypt; 4 Faculty of Medicine, University of Nis, Nis, Serbia; 5 Department of Medicine, National and Kapodistrian University of Athens, Athens, Greece; 6 Faculty of Science, Cairo University, Cairo, Egypt; 7 Faculty of Medicine, Al-Azhar University, Dameitta, Egypt; 8 Faculty of Medicine, Menofia University, Shebin al-Kom, Egypt; 9 Rangaraya Medical College, Kakinada, India; 10 Department of Family and Community Medicine, College of Medicine, King Khalid University, Abha, Saudi Arabia

**Keywords:** Monkeypox, Stigma, Discrimination, Confidentiality, Ethics, Moral issues

## Abstract

**Background:**

Historically, epidemics have been accompanied by the concurrent emergence of stigma, prejudice, and xenophobia. This scoping review aimed to describe and map published research targeting ethical values concerning monkeypox (mpox). In addition, it aimed to understand the research gaps related to mpox associated stigma.

**Methods:**

We comprehensively searched databases (PubMed Central, PubMed Medline, Scopus, Web of Science, Ovid, and Google Scholar) to identify published literature concerning mpox ethical issues and stigma from May 6, 2022, to February 15, 2023. The key search terms used were “monkeypox”, “ethics”, “morals”, “social stigma”, “privacy”, “confidentiality”, “secrecy”, “privilege”, “egoism”, and “metaethics”. This scoping review followed the framework proposed by Arksey and O’Malley in 2005 and was further improved by the recommendations of Levac et al. in 2010.

**Results:**

The search strategies employed in the scoping review yielded a total of 454 articles. We analyzed the sources, types, and topics of the retrieved articles/studies. The authors were able to identify 32 studies that met inclusion criteria. Six of the 32 included studies were primary research. The study revealed that the ongoing mpox outbreak is contending with a notable surge in misinformation and societal stigma. It highlights the adverse impacts of stigma and ethical concerns associated with mpox, which can negatively affect people with the disease.

**Conclusion:**

The study’s findings underscore the imperative need to enhance public awareness; involve civil society; and promote collaboration among policymakers, medical communities, and social media platforms. These collective endeavors are crucial for mitigating stigma, averting human-to-human transmission, tackling racism, and dispelling misconceptions associated with the outbreak.

**Supplementary Information:**

The online version contains supplementary material available at 10.1186/s12910-024-01078-0.

## Introduction

In 1970, the Democratic Republic of the Congo reported the first documented case of mpox. Mpox was diagnosed in a nine-month-old child [1], marking the first recorded cases of human infection in history. Following its initial identification, the virus spread to other regions of Africa, primarily within the tropical rainforest zones. The disease was reported in Cameroon, the Central African Republic, Nigeria, Gabon, Ivory Coast, and South Sudan [2]. The threatening virus was known to be endemic in these regions for five decades [3].

On July 23, 2022, the World Health Organization (WHO) declared a renewed outbreak of mpox, designating it a Public Health Emergency of International Concern (PHEIC) [4]. By May 10, 2023, the International Health Regulation (IHR) determined that the ongoing mpox outbreak no longer posed a PHEIC [5]. Consequently, revised interim recommendations were issued to facilitate the transition towards a long-term strategy for controlling mpox [5]. In May 2023, the Centers for Disease Control and Prevention (CDC) reported 87,314 confirmed mpox cases across 111 countries. Notably, over 90% of these cases emerged in regions traditionally not affected with mpox endemicity—specifically, Europe, Australia, and North America [6, 7]. This indicates significant virus spread beyond its usual geographical boundaries [8]. 

The causative agent of the outbreak, monkeypox virus (MPXV), is a double-stranded DNA virus belonging to the Orthopoxvirus genus, closely related to the smallpox virus. MPXV is capable of infecting both humans and certain animals [9]. Named after monkeys, MPXV was first identified in 1958 in skin lesions of imported monkeys in a Danish laboratory [10]. Human-to-human transmission of MPXV can occur through direct contact with infected skin lesions or mucous membranes, respiratory droplets, and the sharing of contaminated items such as food, bedding, and utensils [11]. The ongoing mpox outbreak that started in 2022 has a wider geographic spread than previous outbreaks, with growing evidence indicating sexual contact as the predominant mode of transmission. Global spread can be attributed to international travel to traditionally endemic regions and participation in large mass gatherings linked to sexual activities [12]. The rapid expansion of human-to-human transmission is indeed amplified within sexual networks, particularly among men who have sex with men (MSM) [13]. Pregnant females have also been diagnosed with mpox during the recent outbreak [14]. Human-to-human transmission can occur within households and children in close contact with infected family members are at risk of contracting the virus. Healthcare professionals (HCPs) who care for sick patients, including those with mpox, are also at risk of contracting the virus if proper infection control protocols are not followed [15]. Fever is typically the initial symptom of mpox, followed by the appearance of a rash after a few days, with concurrent or preceding lymphadenopathy [16]. 

Emerging or re-emerging infectious diseases demand significant attention due to their complex ethical issues. Outbreaks often challenge balancing public health interests with protecting fundamental human rights. Measures like monitoring, isolation, and quarantine may be necessary to control the spread of the disease but must be implemented with respect for individuals’ rights and dignity [17]. 

In the outbreak of mpox in 2022 and according to associated reports, 87.3% of cases were gay, bisexual, and MSM which may fuel the stigma and reduce the acceptance of this highly marginalized community [18, 19]. This situation exhibits striking parallels with the human immune deficiency virus (HIV) epidemic that profoundly affected the lesbian, gay, bisexual, transgender, and queer (LGBTQ) community in the late 1980s and early ’90s [20].

This perspective highlights the dual detrimental effects of attributing the spread of mpox to a specific group: it not only perpetuates stigma against the LGBTQ community but also undermines the recognition of the broader risk to the entire population. As WHO chief Tedros Adhanom Ghebreyesus said “The stigma and discrimination can be as dangerous as any virus and can fuel the outbreak” [21]. Individuals experiencing stigmatized identities encounter heightened vulnerability and discrimination, leading to reluctance to disclose symptoms or seek care [22]. This serves as a barrier to effective prevention, treatment, and containment efforts during outbreaks of this nature [22, 23].

Moreover, HCPs face several delicate ethical dilemmas related to informed consent, patient autonomy, patient confidentiality rights, partner notification, and equity in healthcare [24]. Preventive measures, clinical trials, and research are all subjected to ethical considerations as well. Mandatory vaccination undermines an individual’s autonomy, liberty, and benefit. All researchers and medical professionals must uphold their ongoing commitments to the values of beneficence, fairness, and respect for all people while conducting clinical trials and searching for new antiviral drugs to combat any infectious disease and its spread [25, 26].

Achieving a balance between the libertarian objective of confidentiality and liberty of movement and the utilitarian goal of improving public health in situations involving contagious, fatal, or dangerous diseases presents a complex and challenging ethical question [27]. There is currently no comprehensive literature review that summarizes the ethical concerns and stigma associated with mpox infection. Recognizing this gap, our study was dedicated to filling it by conducting a thorough review of published studies and existing reports. The primary goal was to provide a comprehensive overview of the ethical issues and stigma associated with the mpox outbreak, as well as an examination of associated misinformation. The outcomes of this review will provide insights to inform recommendations for future research, policy development, and ethical guidelines. This approach is designed to address identified gaps and promote ethical decision-making in the context of mpox outbreaks.

### Methodology

This scoping review followed the framework proposed by Arksey and O’Malley [28] and was further improved by the recommendations of Levac et al. [29]. We also adhered to the PRISMA Extension for Scoping Reviews (PRISMA-ScR) developed in 2018 by Tricco et al. [30] and updated in 2020 by Peters et al [31]. (Supplementary 1 file).

This study aimed to.

- Analyze the identified literature to categorize and describe the ethical issues that arose during the mpox outbreak. This includes patient care issues, public health measures, and societal perceptions.
- Investigate and categorize the various types of stigma associated with mpox infection as described in the literature. Investigate how societal attitudes, misinformation, and public perceptions contribute to the stigmatization of mpox patients.
- Examine the role of misinformation in shaping ethical considerations and perpetuating stigma during the mpox outbreak in particular. Recognize the effects of false information on public health responses and individual experiences.

### Database search

Searching for relevant literature published in English was conducted by two authors (AG, RMG) using the following electronic databases: PubMed Central, PubMed Medline, Scopus, Web of Science, Ovid, and Google Scholar. The literature search commenced on February 15, 2023, focusing on published papers from May 6, 2022, onward. This date corresponds to the announcement of the first identified case of mpox. Relevant terms, synonyms and abbreviations were tailored for each database (Supplementary 2 file). The search strategy for PubMed was (“Monkeypox virus“[MeSH Terms] OR “Monkeypox“[MeSH Terms] OR “Monkey Pox“[Text Word] OR “MPX“[Text Word] OR “monkeypox virus*“[Text Word] OR “monkeypoxvirus*“[Text Word] OR “monkey pox virus*“[Text Word]) AND (“Ethics“[MeSH Terms] OR “Morals“[MeSH Terms] OR “Social Stigma“[MeSH Terms] OR “Privacy“[MeSH Terms] OR “Confidentiality“[MeSH Terms] OR “stigma*“[Title/Abstract] OR “moral*“[Title/Abstract] OR “Secrecy“[Title/Abstract] OR “privileg*“[Title/Abstract] OR “confident*“[Title/Abstract] OR “priva*“[Title/Abstract] OR “ethic*“[Title/Abstract] OR “Egoism“[Title/Abstract] OR “metaethic*“[Title/Abstract])

In addition, reference lists, and citation tracking were conducted to identify further related articles. This involved scrutinizing the references of relevant studies, tracking citations, and exploring related articles for eligible publications. Moreover, a supplementary search was performed on gray literature sources (medRxiv and Research Square). In our scoping review methodology, we performed a manual search by systematically reviewing articles pertinent to our research topics in key journals, such as The Lancet, the BMJ, BMC Tropical Medicine and Health, Bioethics, BMC Medical Ethics, and PLOS Neglected Tropical Diseases.

### Study selection

All citations found were imported to an “EndNote” library and duplicate citations were removed. Then, the citations were exported to an Excel sheet file for a two-stage screening process; (a) initial title and abstract screening by two authors independently (A.G, H.A) and (b) full-text screening by another two independent authors (H.E, I.K). The inclusion criteria for studies encompassed all research related to both mpox and ethical issues, published in English and appearing after the first reported case of mpox in May, 6 2022. The agreement between reviewers was 0.83. A third expert reviewer (RMG) resolved any conflicts.

The criteria proposed by Joanna Briggs Institute [32] were followed for our search strategy: Population, Concept, and Context (PCC).

- Population: Any population was included (no restriction for age, sex, race, sexual orientation).
- Concept: This study encompassed all research about both ‘mpox’ and ethical themes, written in English and published after the initial reported case of mpox in the United Kingdom on May 6, 2022.
- Context: All types of research papers were included (original articles, commentaries, brief reports, letters to the editor, opinion articles, short communication, and viewpoints).

Eligible studies for data extraction:

- This criterion ensured that the selected studies provided comprehensive information on the research design, methods, and findings.

### Charting the data

Four reviewers (A.G, H.E, H.A.M, A.G.E) independently retrieved essential data extracted from the eligible articles using the prespecified data extraction form. The extracted data included characteristics of participants (i.e., gender, sexual orientation) and study characteristics (i.e., authors’ last name, year of publication, country, objectives, and study design) and the ethical concerns or stigma related to mpox. The primary outcome of our study was the identification and synthesis of ethical themes pertaining to mpox, derived from the included records (after reviewing relevant studies concerned with the research question of interest). These themes encompassed a range of moral and ethical issues, including managing an infectious individual, misinformation, stigmatized terminology, stigmatized policies, the burden of discrimination within the community, and other pertinent themes observed across the reviewed records. Any disagreement was resolved by consensus or the senior researcher (RMG). The expert panel was consulted as needed, particularly in situations where there was a lack of understanding of the context, or specific terminologies that cannot be understood by the data extractors. Comprising individuals with specialized knowledge and expertise in the subject area (Medical Ethics, infectious diseases, and Tropical Health), the expert panel provided valuable insights and clarification to enhance the comprehensibility of the scoping review.

## Results

### Search results

**Fig. 1:**
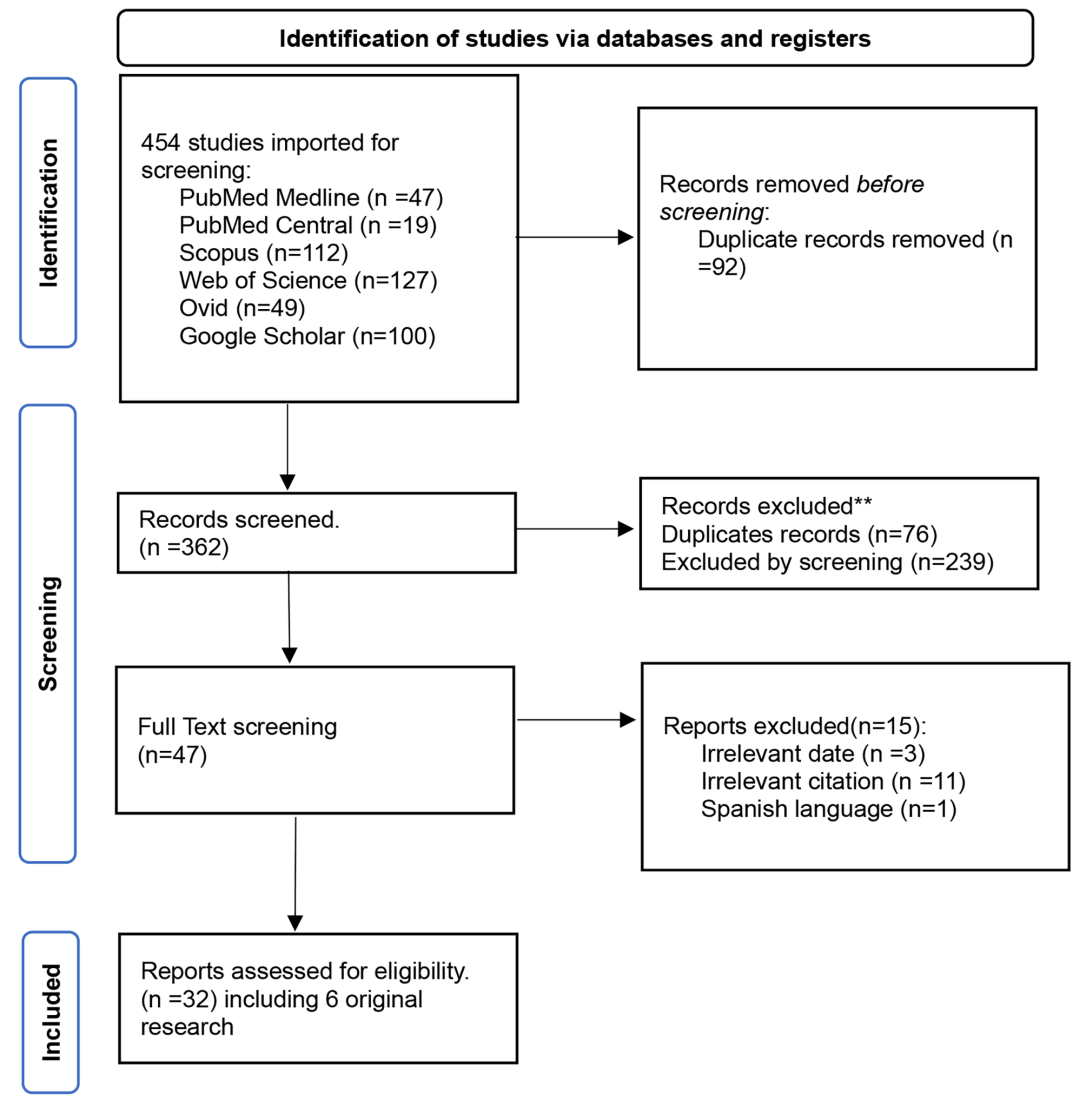
Flow chart of included studies

The search strategies used in the scoping review yielded a total of 454 articles. Among these, 354 articles were identified from various databases. An additional 100 articles were retrieved from Google Scholar. A total of 92 duplicate studies were excluded using Endnote find duplicates function. The remaining 362 citations underwent screening based on their titles and abstracts. During this stage, 76 duplicates and 239 citations were excluded based on their title and abstract, leaving 47 articles for full-text screening. The full-text screening was conducted on these 47 articles, resulting in the exclusion of 15 studies. The reasons for exclusion included irrelevant targeted dates (3 studies), irrelevant citations (11 studies), and one study written in Spanish. Finally, 32 studies were included in the scoping review for further analysis and synthesis. Figure 1.

### Study characteristics

**Fig. 2:**
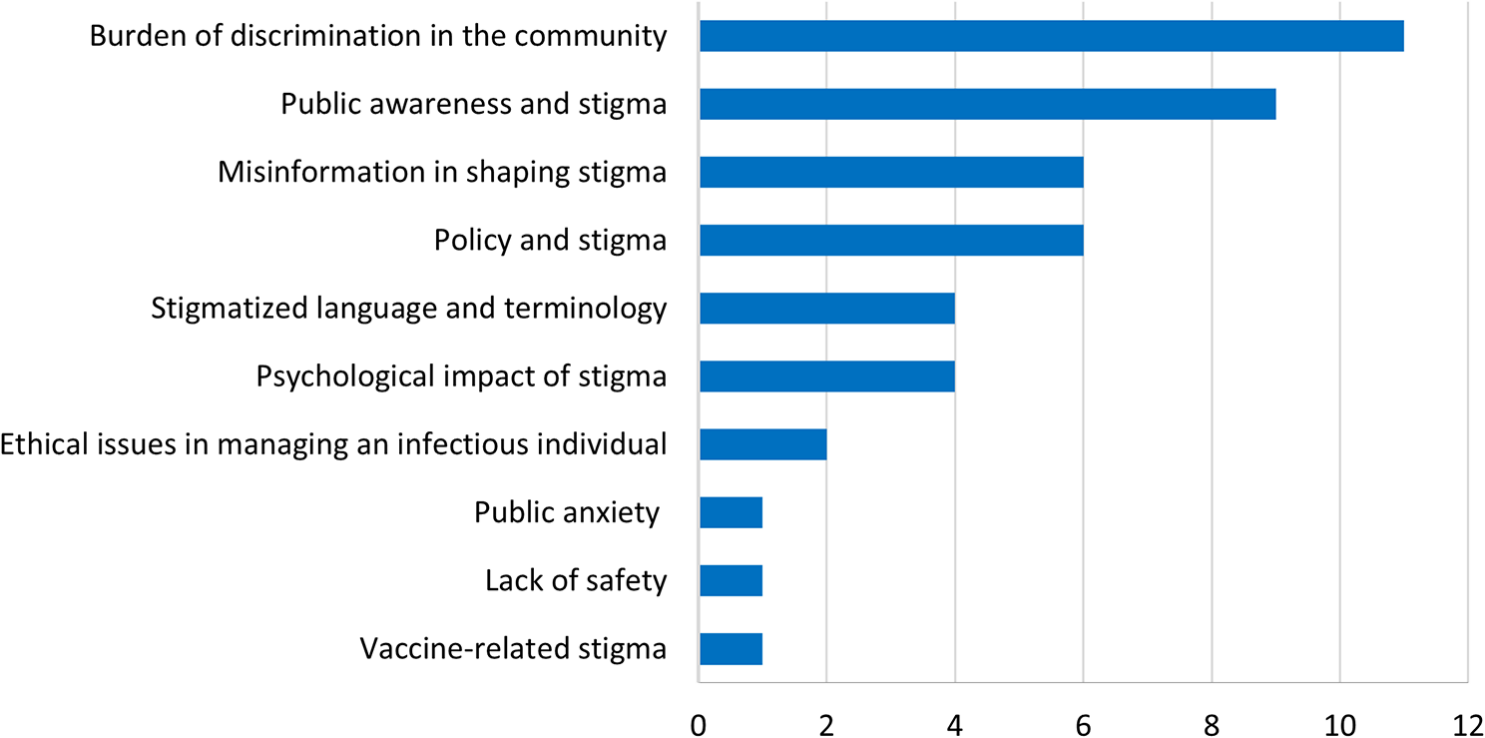
The main themes of the included studies that addressed stigma, discrimination, and ethical concerns related to mpox

Fig.S1. A total of 32 studies were included in the scoping review, they were classified as follows: five letters to the editor [33–37], four commentaries [38–41], four editorials [42–45], three articles [46–48], three opinion articles [49–51], two brief reports [52, 53], two short communication [54, 55], two viewpoints [56, 57], one article info [58], one clinical article [59], one correspondence [60], one mini-review article [61], one news [62], one open letter [63], and one perspective article [64]. Table S1 illustrates the characteristics of the included studies. The following section will discuss highlighted ethical themes in the aforementioned studies. Figure 2.

### Burden of discrimination in the community

Eleven articles addressed the burden of discrimination related to mpox infection. Mungmunpuntipantip [35] discussed the importance of tackling stigma related to mpox to effectively control disease transmission. Shukla et al., [37] highlighted the need for addressing stigma and discrimination towards LGBTQ community, particularly in developing countries like India. W. März et al., [39] addressed the sociopolitical consequences of the mpox outbreak for the gay, bisexual, and MSM, as well as the broader lesbian, gay, bisexual, transgender, queer, and intersex (LGBTQI+) community leading to discrimination and isolation of these marginalized groups. Dsouza et al., [48] collected tweets on mpox stigma among the LGBTQ + community and analyzed its sentiment and content. According to analysis, the LGBTQ + community faces stigma associated with mpox, which may discourage individuals from seeking treatment and may result in untreated infections. Aquino et al., [40] mapped out the unintended centralization of marginalized groups by public health communications, advisories, and policies. In addition to the targeted campaigns that raise conceptual ambiguities and risks attaching a stigma to marginalized groups and mpox. Yang et al., [41]. proposed a measure that utilizes the three stages of the stigma development process and aims to prevent the emergence, progression, and dissemination of stigmatization related to mpox. Kenyon [52] applied Spearman’s correlation to assess the relation between the national incidence of mpox in European countries and the intensity of screening for sexually transmitted diseases (STDs) and a composite indicator of lesbian, gay, bisexual, and transgender and intersex (LGBTI) rights (the Rainbow Index). The report highlighted the stigmatizing attitudes to homosexuality as the causing factor for the reduced utilization of screening tests for STDs and therefore low incidence of mpox reported from the various Eastern European Nations. Ng et al., [54] analyzed the sentiment of the Twitter post towards the outbreak of mpox through unsupervised machine learning that retrieved stigmatization of minority communities. März et al., [56] represented their perspectives on the ethical challenges posed by mpox outbreaks within the LGBTQI + community, highlighting health inequalities, the heightened stress, and fear of further marginalization experienced by this community. Iglesias et al., [63] investigated the social perspective of considering mpox as a sexually transmitted virus. The authors emphasized the need for critical thinking for efficient communication, they also discuss social inequities and highlight the value of social science. Happi et al., [64] propose a novel classification of mpox that is non-discriminatory and non-stigmatizing and aligned with best practices in naming infectious diseases in a way that minimizes unnecessary adverse impacts on countries, geographic regions, economies, and people and that considers the evolution and spread of the virus.

### Public awareness and stigma

Nine publications highlighted the awareness, lessons learned, and stigma associated with mpox outbreak.  Lee & Morling [42] discussed the importance of public awareness campaigns, targeted vaccination strategies for high-risk populations, and robust surveillance systems in preventing stigma. This resonates with De Sousa et al., [43] who insisted on the necessity of inclusive surveillance and health education strategies and decoupling public health intervention from specific affected groups to prevent prejudice and stigma. They emphasized the need to raise public awareness, engage civil society, and improve cooperation between policymakers, the medical community, and social media platforms to prevent stigma and disseminate precise and authoritative information regarding mpox. Islam et al., [36] addressed the crucial role of advocating for public awareness to reduce the global health burden. Mirroring previous outbreaks, Dzobo et al., [44] highlighted the lesson learned from the coronavirus disease 2019 (COVID-19) in implementing education, advocacy, and awareness strategies for reducing stigma and promoting coordinated efforts on a global scale in response to disease outbreaks. Finally, Gonsalves et al., [45] compared the emerging mpox with HIV as both share similarities in the global and domestic response to these outbreaks, highlighting the lack of public awareness and the delay in responding to outbreaks in Africa as well as stigmatizing attitudes. Chang et al., [49] discussed that the lack of public awareness highly promotes stigma that can be eliminated through the widespread distribution of educational resources. Ogunbajo [53], conducted a community initiative for vaccinating black sexual minority men (SMM) in Washington D.C. with mpox vaccines, in addition to a survey for assessing the demographics and health beliefs of participants. The report highlighted that participants had a high level of expected stigmatization for mpox patients, presenting the urgent need for public education and awareness regarding mpox. Raheel et al., [55] discuss the importance of awareness campaigns such as “CDC’S highly successful Let’s Stop HIV Together” that motivate individuals to establish preventive measures and seek healthcare. Using a case-based discussion, Bergman et al., [59] discussed stigma prevention strategies via community awareness and nursing approaches in enhancing awareness among healthcare providers and patient education.

### Policy and stigma

Six studies focused on the policies and stigma.  Chang et al., [49] discussed that policies may encourage discrimination where the implementation of a national action plan is necessary to support the response to stigma during infectious disease outbreaks. W. März et al., [39] highlighted the urgent necessity of increasing policymakers’ awareness regarding the sociopolitical consequences of the mpox outbreak for the gay, bisexual, and MSM, as well as the LGBTQI+ community. Hence, he introduced a policy recommendation to address the mpox outbreak within a comprehensive policy framework to advance LGBTQI + health equality. De Sousa et al., [43] emphasized the need to improve cooperation between policymakers, the medical community, and social media platforms to prevent stigma and disseminate precise and authoritative information regarding mpox. Ng et al., [54] analyzed the sentiment of the Twitter post towards the outbreak of mpox through unsupervised machine learning that retrieved a general lack of faith in public institutions. März et al., [56] represented their perspectives on the ethical challenges posed by mpox outbreaks within the LGBTQI + community, highlighting concerns regarding the neglect of the mpox outbreak by policymakers. Scheffer et al. [57] wrote about their perspective on human rights-based approaches in epidemic responses. They advocate for policies and interventions guided by principles such as equity, inclusion of vulnerable populations, and active participation of affected communities in finding solutions.

### Misinformation in shaping stigma

The association between misinformation and stigma was highlighted in six studies. Farahat et al., [33] focused on the -misinformation on social media that impedes the ability of healthcare experts to communicate effectively. Ju et al., [46] analyzed how the media (Washington Post) is handling both COVID-19 and mpox outbreaks and its role in framing stigma within communities. After stigmatizing China as the origin of COVID-19, the news shifted to stigmatizing Africa with mpox. Moreover, it indirectly labels gays as a special group more susceptible to mpox infection. Alsanafi et al., [47] assessed current disease knowledge among Kuwaiti HCPs and evaluated their attitudes concerning virus emergence conspiracies. The article highlighted the lack of knowledge among HCPs regarding mpox infection, diagnosis, and management. Moreover, the false belief that infection is exclusive to gay leads to discriminatory attitudes and stigmatization towards affected persons. Chang et al., [49] discussed how critical it is for the media to avoid drawing incorrect conclusions from research on mpox in non-endemic areas. Singla & Shen [60] assured that in the majority of countries, social media are unregulated, and the accumulation of false information regarding various epidemics is widespread. When such deceptive and misleading information reaches the public and uninformed individuals, it can create havoc or a new kind of social stigma. Singla et al., [61] published a comprehensive review of existing literature on the biased studies that reported mpox cases in the LGBTQ community and stated that despite the small amount of data regarding the sexual orientation of the patients, the media exacerbates the existing stigma towards the community.

### Psychological impact of stigma

Chang et al., [49] discussed that affected individuals and families are vulnerable to internalized stigma due to anxiety, depression, and suicide ideation, highlighting the importance of mental health support and raising awareness. Sah et al., [50] urged the need to investigate how the stigma associated with mpox impacts the infection’s various differential diagnoses and health effects, particularly mental hygiene, underscoring the impact of mpox on mental health. Infected individuals are more likely to experience mental health issues such as depression and anxiety disorders. März et al., [56] represented their perspectives on the ethical challenges posed by mpox outbreaks within the LGBTQI + community, highlighting stress and fear of further marginalization experienced by this community. Bergman et al., [59] discussed different stigma types experienced by mpox patients including shame feeling, self-blame, fear of judgment, and lack of social support which can lead to depressive symptoms, psychological stress, isolation, and economic consequences.

### Stigmatized language and terminology

Four studies focused on the stigmatized and terminology related to mpox. Islam et al., [36] addressed the crucial role of advocating for avoiding stigmatized language in mpox communication to reduce the global health burden. Furthermore, there is a stigmatization of individuals and communities associated with the name “monkeypox” where comments often labeled it as a “gay disease” OR “monkey disease”. These stigmatizing associations were found to hinder the detection and treatment rates of the disease. In response to these concerns and after consulting with experts, the WHO decided on November 28 to change the name from “monkeypox” to “mpox“ [38]. Also, Taylor [62] discussed the change of mpox name from the old one “monkeypox” after the publication of a letter by over thirty scientists worldwide on June 10th, asking for terminology revision where there is a need to correct the terminology to reduce racism, and stigma and to compact the widespread misinformation. Chang et al., [49] explored how discriminatory language could impede medical response and prevent help-seeking behavior in cases of HIV, COVID-19, and Ebola and currently in mpox. And how critical it is for the media to adopt clearer terms to avoid drawing incorrect conclusions from research on mpox in non-endemic areas.

### Ethical issues in managing an infectious individual

Two studies addressed ethical concerns related to the management of mpox patients. Shrewsbury [51] discussed a certain situation in which the infected person was subjected to blame and shame. Regardless of whether they caught their infection through sexual contact or by encountering a contaminated surface, everyone deserves treatment. HCPs should keep these guidelines in mind and try to be more aware of situations in which they can unintentionally embarrass or assign blame. They should acknowledge that mpox and all other contagious illnesses should be contained and treated with a commitment to unconditional empathy. Iglesias et al., [63] investigated the healthcare consequences of considering mpox as a sexually transmitted virus. The authors emphasized the need for critical thinking for efficient communication.

### Vaccine-related stigma

Mazzagatti et al. [58] addressed the burden of stigma among the already criticized community of bisexuals. It underlines the detrimental effects on patient confidence and intention to take preventive measures, drawing comparisons to the historical stigmatization of persons living with HIV. Because of the concentration only on immunizing high-risk individuals, particularly MSM, “vaccine-related stigma” and restricted access to the vaccine for people who do not frequently visit sexual health clinics are emerging. The article advises getting rid of this stigma by making vaccination available to all sexually active bisexuals and identifying each person’s risk factors through interviews or questionnaires. Additionally, it emphasizes how crucial it is to safeguard private information obtained during vaccinations and offer shots outside of sexual health clinics. The article’s conclusion highlights the importance of timely and precise communication while avoiding ambiguous information that can feed stigma against the LGBTQ + community.

### Public anxiety

Lee & Morling [42] discussed the impact of public anxiety from unfamiliar emerging diseases, which contributes to germ-induced panic accompanied by the stigmatization of the condition and detrimental psychological consequences for both affected individuals and communities.

### Lack of safety

Ng et al., [54] analyzed the sentiment of the Twitter post towards the outbreak of mpox through unsupervised machine learning. This approach retrieved general concerns regarding safety, reflecting the public’s fear that the increasing number of mpox cases and the WHO declaration it a PHEIC resembles the early stages of the COVID-19 pandemic. Although mpox is not as transmissible as COVID-19 and a vaccine is available, the risk of cross-border transmission persists, particularly with increasing international travel and interconnectedness. Therefore, the author emphasized the importance of providing accurate and timely information on mpox.

Only six studies were deemed suitable for data extraction, including three articles [46–48], two brief reports [52, 53], and one short communication [54]. Table 1 These studies encompassed a total of 418,569 Twitter posts, 896 HCPs, 127,000 European MSM survey, 188 SMM in the United States of America (USA), and 71 online news reports [46–48, 52–54]. The inclusion of these different types of publications allowed for a comprehensive exploration of the ethical issues related to the outbreak of mpox infection and provided diverse perspectives and insights into the topic at hand.

### Study design

Of the eligible studies, two studies incorporated content analysis of Twitter posts [48, 54], one study employed content analysis of The Washington Post’s Online News [46], one was a cross-sectional study for HCPs in Kuwait [47], one was an ecological analysis of European men who have sex with men internet survey in 40 countries [52], and one cross-sectional study of SMM in the USA [53].

### The main ethical issues

The ethical issues related to human mpox have been observed at various levels, including country, institute, community, and individual. At the country level, countries with more stigmatizing attitudes towards homosexuality tend to have lower reported rates of screening for STDs and a lower incidence of mpox [52]. At the institute level, news media (The Washington Post) has been found to construct differential stigmas that indirectly label gays as being more likely to be infected with mpox, leading to increased stigma and discrimination towards them, labeling African countries as the “typical source of mpox”, and identifying the COVID-19 outbreak in China being deemed as a cause for alarm while the mpox cases spreading in the USA being regarded as not being a significant concern [46]. At the community level, it has been observed that the community of LGBTQ + on Twitter has been affected in a way that they refrain from any public health measures about mpox [48]. Content analysis of public Twitter posts has also revealed stigma towards LGBTQ and racial minority communities, lack of faith in institutions, and governmental efforts to contain mpox and misinformation about the infection as a political conspiracy [54]. At the individual level, certain observations have been made regarding ethical issues related to mpox. For instance, in Kuwait, there is a higher prevalence of conspiracy beliefs regarding emerging virus infections among certain groups. Females, individuals with lower knowledge about mpox, and those who agreed or had no opinion regarding the exclusivity of mpox incidence among gays were found to be more likely to embrace conspiracy beliefs [47]. In the USA, particularly among bisexuals, a significant proportion of respondents (ranging from 13 to 31%) reported the belief that various people in their lives judge them if they were to contract mpox. Additionally, 35% of respondents believed they would be blamed for their infection, and 51% believed that others would assume they were sexually promiscuous if they acquired mpox [53].

**Table 1:** Studies that addressed ethical concerns related to mpox

Author and Year	Objective	Study design	Country (Study setting)	Sample Size	Findings
Ju W et al. (2023) [46]	The way the media “Washington post-WP” is presenting health crises COVID-19 and mpox infections, and its role in framing stigma among affected communities.	Qualitative content analysis	USA	15 reports of mpox and 56 reports of COVID-19, making the total 71 online news reports.	Formulation of different stigmas during pandemics, by the early COVID-19 and mpox outbreaks: After stigmatizing China as the origin of COVID-19, the news shifted to stigmatizing Africa. Moreover, it indirectly labels gays as a special group more susceptible to mpox infection. Panic and fear from the spread of COVID-19 infection in China, and the opposite reaction concerning mpox spreading in the United States.
Alsanafi M et al. (2022) [47]	The study assessed HCPs’ knowledge, trust in disease diagnosis and management, and belief in emerging viral infections.	Cross-sectional study	Kuwait (Web-based survey)	896 hHCPs: physicians, dentists, nurses, pharmacists, and medical technicians	A lack of knowledge among HCPs regarding mpox infection, diagnosis, and management. False belief that infection is exclusive to homosexuals, results in an attitude of discrimination and stigmatization towards affected persons.
Dsouza VS et al. (2022) [48]	To identify and map the mpox stigma within the (Lesbian, gay, bisexual, transgender, queer, and more) LGBTQ + community on Twitter	Content analysis	India (Online Content analysis using Twitter)	A total of 66,387 tweets	This study employed a stigma communication model to map and estimate the degree of mpox stigma within the LGBTQ + community on Twitter. The LGBTQ + group faces stigma associated with mpox, which may discourage individuals from seeking treatment and may result in untreated infections.
Kenyon C (2022) [52]	The purpose of this study was to investigate the association between the national incidence of mpox and the extent of screening for sexually transmitted diseases (STDs) and LGBTQ rights.	Brief Report of ecological analysis	40 countries participating in the European men who have sex with men (Online Survey)	127,000 European bisexual	Lower reported rates of STDs screening and a lower occurrence of mpox were seen in nations with more discriminatory views towards homosexuals.
Ogunbajo A et al. (2022) [53]	To assess the demographics and health beliefs of black gay, bisexual, and other sexual minority men who received a mpox vaccination.	Cross-sectional study	USA (A Community-based intervention)	178 Black African American, Gay/homosexual 146 (82.0)	Participants had a high socioeconomic position, a high amount of anticipated mpox stigma, and were generally suspicious of mpox, driven by false beliefs.
Ng QX et al. (2022) [54]	Application of machine learning in analysis of Twitter posts for assessing public sentiment towards the global outbreak of mpox	Content Analysis	Singapore (modeling and Thematic analysis of Twitter)	352, 182 Twitter posts	The analysis of Twitter data regarding public sentiment about the global outbreak of mpox retrieves main three themes. These include concerns about safety, stigmatization of minority communities, and a general lack of faith in public institutions.

USA: United States of America; STDs: Sexually transmitted diseases; COVID-19: Coronavirus disease 2019; mpox: Monkeypox; HCPs: Health care professionals; LGBQ: lesbian, gay, bisexual, transgender, and queer

## Discussion

Discrimination and stigma associated with any disease, including mpox, are never acceptable. They can have a serious impact on health outcomes and undermine outbreak response efforts by making people hesitant to come forward or seek care. This increases the risk of transmission, both within and beyond the most affected communities [65]. This scoping review aimed to identify and outline the primary ethical challenges associated with the outbreak of mpox. This review included 32 studies. Out of these studies, only six were suitable for data extraction, including three articles [46–48], two brief reports [52, 53], and one short communication [54]. These studies covered various topics such as Twitter posts, HCPs, MSM surveys, and online news reports. The study designs varied among the eligible studies, including content analysis of Twitter posts, analysis of online news, cross-sectional studies, ecological analysis, and community-based interventions. These different approaches provided a comprehensive understanding of the ethical issues associated with the outbreak of mpox infection.

### The main findings of the study

The insights into the mpox outbreak and the resulting stigma paint a complicated picture. Misinformation on social media emerges as a significant barrier to effective communication among healthcare experts, emphasizing the need for a more coordinated response. Policy recommendations, lessons learned from previous epidemics, and assessments of media articles all emphasize the importance of clear messaging, public education, and the use of empathetic language. Ethical issues emerge at multiple levels, necessitating a concerted effort to monitor social media, address discriminatory language, and recognize the impact on marginalized communities. The decision to rename the virus “mpox” rather than “monkeypox” reflects a strategic move to reduce stigma. Notably, themes of targeted testing, vaccination initiatives, and stigma reduction take center stage, particularly among the LGBTQI + community, emphasizing the need for a comprehensive and compassionate approach to navigating the challenges posed by the mpox outbreak.

### Misinformation and social media during an infectious outbreak

Infectious disease epidemics are often accompanied by scientific uncertainty, social and institutional instability, and a general atmosphere of fear and distrust. The media plays a significant role in amplifying these reactions. Misinformation is the dissemination of inaccurate or occasionally false statements that contradict the scientific community’s established understanding. Disinformation, on the other hand, can be defined as the intentional dissemination of false information with the goal of achieving secondary benefits, whether financial, political, or a combination of both [66]. In the era of social media, both misinformation and disinformation raise significant concerns, particularly in the context of spreading knowledge related to infectious diseases [67]. The current study highlights the issue of misinformation surrounding the mpox outbreak, emphasizing the need for public awareness, civil society engagement, and cooperation between policymakers, medical communities, and social media platforms to counteract stigma and combat human-to-human transmission, and racism. The negative impact of rumors and misinformation was previously addressed during COVID-19 [67–69]. Unverified COVID-19 rumors can undermine preparedness, lead to incorrect treatments, and diminish healthcare workers’ agency. Social stigma can hinder active participation in public health measures [69]. Individuals can be empowered by media literacy programs to distinguish between reliable and misleading sources while fact-checking initiatives to ensure the timely correction of inaccuracies. Support and training for healthcare workers are critical in navigating rumors and social stigma, with an emphasis on trust-building strategies. International cooperation, drawing on lessons learned at COVID-19, can strengthen the global response to misinformation. Finally, encouraging ethical communication, transparent reporting, and responsible information sharing contribute to a more informed and resilient society in the face of infectious disease epidemics.

### Improving public awareness and increasing health literacy

Health literacy refers to an individual’s ability to access and comprehend health-related information, allowing them to make informed decisions about their health. This includes the ability to effectively seek, understand, and apply health information, allowing individuals to navigate healthcare systems, engage in preventive measures, and make health-related decisions [70]. Advocating for public awareness, emphasizing preventive measures, and avoiding stigmatized language in mpox communication all play critical roles in mitigating the outbreak’s global health burden [71]. Individuals can make informed decisions about protective measures by raising public awareness and lowering the risk of transmission. Emphasizing preventive measures, such as mpox testing and vaccination, can help to break the chain of infection. Interestingly, despite the proven efficacy and effectiveness of the mpox vaccine [72], there are notable high rates of vaccination hesitancy observed among the general population and HCPs despite the high vaccine effectiveness [72]. This phenomenon may be attributed to a lack of trust in vaccination and potential issues related to health illiteracy [73, 74]. This could be due to a lack of trust in vaccinations and low health literacy. A study conducted by Alsanafi et al. [47] in 2022 highlighted that a significant percentage (20.4%) of HCPs held incorrect beliefs, such as assuming that mpox is exclusively associated with MSM. The study also found that the degree of education and occupation played a role in shaping these beliefs, with medical technicians and allied health professionals demonstrating lower knowledge compared to physicians and pharmacists. It is important to emphasize that mpox should not be incorrectly labeled as a “gay disease.” Sexual orientation does not determine an individual’s risk of infection. Understanding the actual modes of transmission is crucial in dispelling such misconceptions. By promoting accurate information and education, we can correct misunderstandings and challenge stereotypes associated with mpox [75, 76]. So that public health campaigns should focus on disseminating knowledge about mpox transmission, emphasizing the importance of hygiene practices, early detection, and seeking appropriate medical care. These efforts can help reduce stigma, increase awareness, and ensure that individuals and communities are equipped with the correct information to make informed decisions regarding their health and the prevention of mpox transmission. Additionally, avoiding stigmatized language is critical in creating a supportive environment that encourages people to seek information and healthcare without fear of being judged. This approach not only improves community cooperation, but also aids in the dispelling of myths and lowers the overall impact of stigma on affected individuals. Overall, these advocacy efforts are critical components of a comprehensive global strategy to address and control the mpox outbreak. To reduce the harm caused by stigma and discrimination, we must actively reflect on and act on our language, behavior, and intentions as individuals and on the policies and practices of organizations, such as healthcare facilities and media outlets [65]. 

### Stigma/discrimination is the main ethical concern in the literature

Infectious disease outbreaks often trigger stigma [77]. Stigma involves the withholding of social acceptance from an individual or group due to a trait perceived as discrediting by their community or society. Stigma proportionality refers to the degree to which stigma is justified or proportional in relation to the actual risks or characteristics associated with a particular group or condition. This broad concept encompasses the cognitive or emotional support of negative stereotypes, known as prejudice; negative behavioral expressions, termed discrimination; and the unjustifiable avoidance or neglect of affected individuals from a medical perspective [78]. The research papers included in this review primarily emphasize the persistent issue of stigma, discrimination, and social disapproval faced by individuals affected by mpox. The stigma and prejudice associated with mpox have significant consequences for individuals living with the disease as well as those connected to infected individuals. Moreover, stigma linked to infectious disease outbreaks diminishes the chances of affected individuals to achieve physical, social, and psychological well-being, thereby exacerbating social and health disparities [79]. One of the detrimental effects of stigma is that it drives individuals to hide their illness, leading to the hidden and undetected spread of the virus. Additionally, stigma can impede efforts to control disease outbreaks by fueling fear, diminishing the uptake of preventive measures (including vaccination), discouraging health-seeking behavior such as seeking testing and treatment, and reducing adherence to care [80]. This stigma extends to partners, children, and caregivers who may face unfair judgment and mistreatment simply for their association with infected individuals. The resulting stigma and discrimination further exacerbate the emotional and psychological distress experienced by those affected by mpox [50]. Specifically, stigma associated with COVID-19 and Ebola has been identified as a significant predictor of severe psychological distress, depression, anxiety, and symptoms of posttraumatic stress disorder [79]. Moreover, public health interventions implemented like quarantine, contact tracing, and vaccination during outbreaks can influence the stigma associated with a disease [81–83]. While evidence of exacerbated stigma may not entirely negate the efficacy of these public health measures, it underscores the importance of considering and minimizing inadvertent social consequences wherever feasible. This pattern of behavior is not unique to the current situation but has been observed in the past with the emergence of novel pathogens. Throughout history, human communities have demonstrated a tendency to isolate, stigmatize, or avoid groups of individuals perceived as having qualities or traits that are considered disagreeable or potentially harmful to others [84–86]. Gonsalves et al. [45] aptly coined the phrase “Déjà vu All Over Again?” to describe the similarities between the stigma surrounding the announcement of mpox and the stigma experienced in previous infectious disease outbreaks. This comparison draws parallels to the panic and discrimination that emerged during the early years of the AIDS epidemic. During that time, individuals living with HIV/AIDS faced stigmatization, particularly those who were confirmed to have the infection, as well as the “four Hs” identified by the CDC: homosexuals, heroin addicts, hemophiliacs, and Haitians [87]. By acknowledging these recurring patterns, we can work towards breaking the cycle of stigmatization and fostering a more inclusive and supportive society for individuals affected by infectious diseases. Addressing the ethical challenges posed by stigma during infectious disease outbreaks requires a multifaceted approach. By promoting education, sensitivity in public health interventions, empathy, and advocacy for equitable policies, we can work towards fostering a society that upholds the rights and dignity of all individuals, thereby mitigating the adverse effects of stigma.

### Actions to mitigate the stigma and discrimination associated with mpox

To overcome and combat negative attitudes and harmful language directed at mpox patients, WHO has taken multiple steps. In December 2022, public advice was published, by the WHO concerned with stigma and discrimination, targeting all organizations (governmental and non-governmental), health practitioners, authorities, as well as media dealing with the outbreak [88]. Recently, a policy brief was released on the 23 of July 2023, offering guidance on critical ethical issues that have arisen in the context of the mpox outbreak response. Three key domains were emphasized: stigma /discrimination, the availability and distribution of medical services, and the importance and responsibility of scientifically based evidence [89]. WHO recently released its public health advice on understanding, preventing, and addressing stigma and discrimination related to mpox, which provides information on the potential impact of stigma and recommends language and actions to counter stigmatizing attitudes and discriminatory behaviors and policies [89]. 

### Points of strength and limitations

This scoping review is unique in its contribution as it is the first attempt to systematically analyze the existing published evidence regarding ethical dilemmas and discrimination related to mpox. By mapping out the identified moral themes, the review provides valuable insights into the current understanding of ethical challenges in mpox and identifies areas that require further exploration. Second, there is a paucity of articles addressing ethical issues related to mpox, highlighting the importance of this review to identify research gaps in the existing literature. However, this review has some limitations that should be addressed. First, the review primarily focused on the theme of stigma and discrimination associated with mpox. Other ethical principles were not extensively explored. This suggests a need for further research to assess and address a broader range of ethical issues related to mpox outbreaks. It would be of paramount value to probe both the community and HCPs’ perception of ethical values and norms surrounding the mpox infection. Second, most of the included studies originated from Western countries, neglecting the main origin of the infection in African regions. This geographical bias emphasizes the importance of conducting research in the affected areas to comprehensively understand the ethical challenges specific to those contexts. Furthermore, the makeup of the expert panel does not appear to contain persons chosen for their association with the group most affected by this outbreak, which may potentially limit the mitigation of epistemological violence and the comprehensiveness of perspectives. Another limitation was that studies on marginalized groups including rural communities and low-resource environments, which are disproportionately impacted by infectious diseases like mpox were noticeably lacking. Finally, the search string employed in the scoping review included relevant terms related to the mpox virus and ethics. While comprehensive, the approach has certain limitations. These include potential trade-offs between sensitivity and specificity, variability in terminology, the risk of publication bias towards articles published in certain journals and/or indexed in certain databases, a lack of consideration for temporal variations, language bias towards English, conceptual complexity with terms like “egoism” and “metaethics,” and potential disparities in database recognition of search terms.

## Conclusions

Despite the declaration that the multi-country outbreak of mpox is no longer a PHEIC, the possibility of the reemergence of mpox remains due to several interconnected factors. Among these factors, the stigma and ethical issues associated with the disease play a significant role. The stigma surrounding mpox can have detrimental effects on various aspects of the disease. It can lead to individuals avoiding seeking care and assistance. Ethical issues arising from mpox, such as discrimination, privacy concerns, access to healthcare, and the conduct of clinical and vaccine studies, further contribute to the challenges in effectively addressing the disease. Consequently, addressing stigma and ethical issues related to mpox is crucial in preventing its resurfacing and ensuring effective control measures. By promoting awareness, education, and understanding about disease and combating stigmatizing attitudes, we can create an environment that encourages individuals to seek timely care and support. Additionally, addressing ethical concerns through appropriate policies, guidelines, and interventions can help protect the rights and well-being of individuals affected by mpox.

### Electronic supplementary material

Below is the link to the electronic supplementary material.

Supplementary Material 1: Figure (S1): Bar chart of the included studies type.

Supplementary Material 2

## Data Availability

Data is available upon request by contacting the second author.
