# Supplementary material for: Ethical considerations during Mpox Outbreak: a scoping review
Source: BMC Med Ethics. 2024 Jul 22;25:79. doi: 10.1186/s12910-024-01078-0 (PMC11265031; doi:10.1186/s12910-024-01078-0)
Supplement: Supplementary file 2 — Supplementary Material 2 [file 12910_2024_1078_MOESM2_ESM.docx]

Supplementary material

**Table S1: Characteristics of included studies**

| Author, Year | Type of reference | Journal name | Title |
| --- | --- | --- | --- |
| Ju, 2023 [46] | Article | International Journal of Environmental Research & Public Health | Stigmatizing Monkeypox and COVID-19: A Comparative Framing Study of The Washington Post's Online News |
| Alsanafi, 2022 [47] | Article | Pathogens | Monkeypox Knowledge and Confidence in Diagnosis and Management with Evaluation of Emerging Virus Infection Conspiracies among Health Professionals in Kuwait |
| Dsouza, 2022 [48] | Article | Dialogues in Health | A sentiment and content analysis of tweets on monkeypox stigma among the LGBTQ+ community: A cue to risk communication plan |
| Mazzagatti, 2022 [58] | Article info | Public Health in Practice | Monkeypox vaccine-related stigma |
| Ogunbajo, 2022 [53] | Brief report | Journal of Urban Health | Demographics and Health Beliefs of Black Gay, Bisexual, and Other Sexual Minority Men Receiving a Mpox Vaccination in the United States |
| Kenyon, 2022 [52] | Brief report | Epidemiologia (Basel) | Is Monkeypox Being Underdiagnosed in Countries with More Stigmatizing Attitudes Towards MSM with Men? A Simple Ecological Analysis |
| Bergman, 2022 [59] | Clinical article | Journal of the Association of Nurses in AIDS Care | Combating Stigma in the Era of Monkeypox - Is History Repeating Itself? |
| Damaso, 2023 [38] | Commentary | The Lancet Regional Health | Phasing out monkeypox: Mpox is the new name for an old disease |
| März,2022 [39] | Commentary | The Lancet Regional Health | Monkeypox, stigma, and public health |
| Aquino, 2022 [40] | Commentary | BMJ Global Health | Monkeypox and the Legacy of Prejudice in targeted public health campaigns |
| Yangm 2022[41] | Commentary | Infection and Drug Resistance | Combating Stigma and Health Inequality of Monkeypox: Experience from HIV |
| Singla, 2022 [60] | Correspondence | International Journal of Surgery | Monkeypox-a global emergency: What nations should learn from recent COVID-19 pandemic? –Correspondence |
| Lee, 2022 [42] | Editorial | Public Health in Practice | The global monkeypox outbreak: Germ Panic, stigma, and emerging challenges |
| Sousa,2022 [43] | Editorial | Revista Brasileira De Enfermagem | Monkeypox: between precision public health and stigma risk |
| Dzobo, 2022 [44] | Editorial | Public Health Practices (Oxf) | Stigma and public health responses: Lessons learned from the COVID-19 pandemic to inform the recent monkeypox outbreak |
| Gonsalves,2022 [45] | Editorial | Journal of Urban Health | Déjà vu all over again? Emergent monkeypox, delayed responses, and stigmatized populations |
| Farahat, 2022 [33] | Letter to the editor | BMC Tropical Medicine and Health Journal | Infodemic and the Fear of Monkeypox: Call for Action |
| Manirambona, 2023 [34] | Letter to the editor | Journal of Medical Virology | Monkeypox among MSM with men in Africa: The need for testing and vaccination beyond the stigma |
| Mungmunpuntipantip, 2022 [35] | Letter to the editor | Journey of the Association of Nurses in AIDS Care | Stigma and Monkeypox: Correspondence |
| Islam et al , 2022 [36] | Letter to the editor | International Journal of Health Planning and Management | Monkeypox outbreak – No panic and stigma; Only awareness and preventive measures can halt the pandemic turn of this epidemic infection |
| Shukla, 2023 [37] | Letter to the editor | Indian Journal of Psychological Medicine | Stigma, Discrimination, and Psychological Distress among the LGBTQ Community in Times of Monkeypox Outbreak—A Wake-up Call |
| Singla, 2023 [61] | Mini-review article | Indonesian Global Journal of Pharmaceutical Sciences | Biased studies and sampling from LGBTQ communities created a next-level social stigma in monkeypox: a PHEIC |
| Taylor, 2022 [62] | News | BMJ | Monkeypox: WHO to rename disease to prevent stigma |
| Iglesias, 2022 [63] | Open letter | Wellcome Open Research | Is monkeypox an STI? The societal aspects and healthcare implications of a key question |
| Chang, 2022 [49] | Opinion article | Tropical Medicine International Health. | Monkeypox outbreak: Preventing another episode of stigmatization |
| Sah et al, 2022 [50] | Opinion article | Frontiers in Public Health | Stigma during the monkeypox outbreak |
| Shrewsbury, 2022 [51] | Opinion article | BMJ | Blame and shame are harming our response to monkeypox |
| Happi, 2022 [64] | Perspective article | Plos Biology | Urgent need for a non-discriminatory and non-stigmatizing nomenclature for monkeypox virus |
| QX NG, 2022 [54] | Short communication | Public Health Journal | Public sentiment on the global outbreak of monkeypox: an unsupervised machine learning analysis of 352,182 Twitter posts |
| Raheel et al, 2022 [55] | Short communication | Annals of Medicine and Surgery | Monkeypox and spillover effects: Stigmas, solutions, and strategies |
| Marz, 2022[56] | Viewpoint | Swiss Medical Weekly | Monkeypox, bioethics, and the LGBTQI+ community |
| Scheffer et al , 2022 [57] | Viewpoint | The Lancet Regional Health – Americas | Monkeypox in Brazil between stigma, politics, and structural shortcomings: have we not been here before? |

PHIC: public health emergency of international concern; COVID-19: Coronavirus disease 2019; mpox: Monkeypox; LGBQ: lesbian, gay, bisexual, transgender, and queer

**
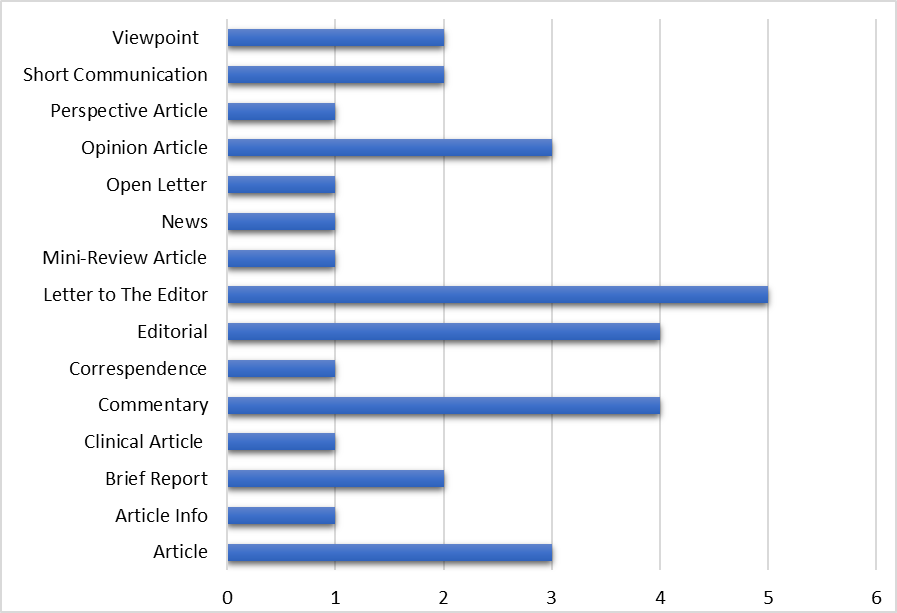
**

**Figure (S1): Bar chart of the included studies type.**

Database search

| Database | Number of citations |
| --- | --- |
| PubMed Medline | 47 |
| PubMed Central | 19 |
| Scopus | 112 |
| WOS | 127 |
| Ovid | 49 |
| Google Scholar | 100 |
| Total | 454 |

**PubMed 47**

("Monkeypox virus"[MeSH Terms] OR "Monkeypox"[MeSH Terms] OR "Monkey Pox"[Text Word] OR "MPX"[Text Word] OR "monkeypox virus*"[Text Word] OR "monkeypoxvirus*"[Text Word] OR "monkey pox virus*"[Text Word]) AND ("Ethics"[MeSH Terms] OR "Morals"[MeSH Terms] OR "Social Stigma"[MeSH Terms] OR "Privacy"[MeSH Terms] OR "Confidentiality"[MeSH Terms] OR "stigma*"[Title/Abstract] OR "moral*"[Title/Abstract] OR "Secrecy"[Title/Abstract] OR "privileg*"[Title/Abstract] OR "confident*"[Title/Abstract] OR "priva*"[Title/Abstract] OR "ethic*"[Title/Abstract] OR "Egoism"[Title/Abstract] OR "metaethic*"[Title/Abstract])

**PubMed Central 19**

("Monkeypox virus"[MeSH Terms] OR "Monkeypox"[MeSH Terms] OR "Monkey Pox"[Text Word] OR "MPX"[Text Word] OR "monkeypox virus*"[Text Word] OR "monkeypoxvirus*"[Text Word] OR "monkey pox virus*"[Text Word]) AND ("Ethics"[MeSH Terms] OR "Morals"[MeSH Terms] OR "Social Stigma"[MeSH Terms] OR "Privacy"[MeSH Terms] OR "Confidentiality"[MeSH Terms] OR "stigma*"[Title/Abstract] OR "moral*"[Title/Abstract] OR "Secrecy"[Title/Abstract] OR "privileg*"[Title/Abstract] OR "confident*"[Title/Abstract] OR "priva*"[Title/Abstract] OR "ethic*"[Title/Abstract] OR "Egoism"[Title/Abstract] OR "metaethic*"[Title/Abstract])

**Scopus 112**

( TITLE-ABS-KEY ( "Monkeypox virus" OR "Monkeypox" OR "Monkey Pox" OR "MPX" OR "monkeypox virus*" OR "monkeypoxvirus*" OR "monkey pox virus*" ) AND TITLE-ABS-KEY ( "Ethics" OR "Morals" OR "Social Stigma" OR "Privacy" OR "Confidentiality" OR "stigma*" OR "moral*" OR "Secrecy" OR "privileg*" OR "confident*" OR "priva*" OR "ethic*" OR "Egoism" OR "metaethic*" ) )

**WOS 127**

TS=("Monkeypox virus" OR "Monkeypox" OR "Monkey Pox" OR "MPX" OR "monkeypox virus*" OR "monkeypoxvirus*" OR "monkey pox virus*") AND **TS=("Vaccines" OR "Immunization" OR "vaccin*" OR "immuni*" OR "inocula*") AND TS"Ethics" OR "Morals" OR "Social Stigma" OR "Privacy" OR "Confidentiality" OR "stigma*" OR "moral*" OR "Secrecy" OR "privileg*" OR "confident*" OR "priva*" OR "ethic*" OR "Egoism" OR "metaethic*")**

**Ovid 49**

("Monkeypox virus" OR "Monkeypox" OR "Monkey Pox" OR "MPX" OR "monkeypox virus*" OR "monkeypoxvirus*" OR "monkey pox virus*") AND **("Vaccines" OR "Immunization" OR "vaccin*" OR "immuni*" OR "inocula*") AND TS"Ethics" OR "Morals" OR "Social Stigma" OR "Privacy" OR "Confidentiality" OR "stigma*" OR "moral*" OR "Secrecy" OR "privileg*" OR "confident*" OR "priva*" OR "ethic*" OR "Egoism" OR "metaethic*")**

**Google Scholar 100**

"Monkeypox virus"|"Monkeypox"|"Monkey Pox"|"MPX"|"monkeypox virus*"|"monkeypoxvirus*"|"monkey pox virus*" "Ethics"|"Morals"|"Social Stigma"|"Privacy"|"Confidentiality"|"stigma*"|"moral*"|"Secrecy"|"privileg*"|"confident*"|"priva*"|"ethic*"|"Egoism"|"metaethic*"
